# Development and validation of a nomogram to predict the 30-day mortality risk of patients with intracerebral hemorrhage

**DOI:** 10.3389/fnins.2022.942100

**Published:** 2022-08-10

**Authors:** Jianyu Zou, Huihuang Chen, Cuiqing Liu, Zhenbin Cai, Jie Yang, Yunlong Zhang, Shaojin Li, Hongsheng Lin, Minghui Tan

**Affiliations:** ^1^Department of Orthopaedics, The First Affiliated Hospital of Jinan University, Guangzhou, China; ^2^Department of Rehabilitation, The First Affiliated Hospital of Jinan University, Guangzhou, China; ^3^Department of Nursing, The First Affiliated Hospital of Jinan University, Guangzhou, China

**Keywords:** intracerebral hemorrhage, MIMIC III database, prognosis, nomogram, mortality

## Abstract

**Background:**

Intracerebral hemorrhage (ICH) is a stroke syndrome with an unfavorable prognosis. Currently, there is no comprehensive clinical indicator for mortality prediction of ICH patients. The purpose of our study was to construct and evaluate a nomogram for predicting the 30-day mortality risk of ICH patients.

**Methods:**

ICH patients were extracted from the MIMIC-III database according to the ICD-9 code and randomly divided into training and verification cohorts. The least absolute shrinkage and selection operator (LASSO) method and multivariate logistic regression were applied to determine independent risk factors. These risk factors were used to construct a nomogram model for predicting the 30-day mortality risk of ICH patients. The nomogram was verified by the area under the receiver operating characteristic curve (AUC), integrated discrimination improvement (IDI), net reclassification improvement (NRI), and decision curve analysis (DCA).

**Results:**

A total of 890 ICH patients were included in the study. Logistic regression analysis revealed that age (OR = 1.05, *P* < 0.001), Glasgow Coma Scale score (OR = 0.91, *P* < 0.001), creatinine (OR = 1.30, *P* < 0.001), white blood cell count (OR = 1.10, *P* < 0.001), temperature (OR = 1.73, *P* < 0.001), glucose (OR = 1.01, *P* < 0.001), urine output (OR = 1.00, *P* = 0.020), and bleeding volume (OR = 1.02, *P* < 0.001) were independent risk factors for 30-day mortality of ICH patients. The calibration curve indicated that the nomogram was well calibrated. When predicting the 30-day mortality risk, the nomogram exhibited good discrimination in the training and validation cohorts (C-index: 0.782 and 0.778, respectively). The AUCs were 0.778, 0.733, and 0.728 for the nomogram, Simplified Acute Physiology Score II (SAPSII), and Oxford Acute Severity of Illness Score (OASIS), respectively, in the validation cohort. The IDI and NRI calculations and DCA analysis revealed that the nomogram model had a greater net benefit than the SAPSII and OASIS scoring systems.

**Conclusion:**

This study identified independent risk factors for 30-day mortality of ICH patients and constructed a predictive nomogram model, which may help to improve the prognosis of ICH patients.

## Background

Intracerebral hemorrhage (ICH) refers to primary, spontaneous, and non-traumatic hemorrhage that occurs in the brain parenchyma (Morotti and Goldstein, 2016; Watson et al., 2022). It is a common and the most severe hemorrhagic stroke syndrome, with a 30-day mortality rate higher than 40%, and most survivors are severely disabled (An et al., 2017). More than 2.8 million people worldwide reportedly die each year due to ICH (Schrag and Kirshner, 2020). ICH usually occurs in small arterioles affected by cerebral small vessel disease (SVD) (Hostettler et al., 2019). It is generally accepted that deep perforator arteriopathy (also termed hypertensive arteriopathy or arteriolosclerosis) (Hostettler et al., 2019; Schreiber et al., 2020) and cerebral amyloid angiopathy (CAA) (Banerjee et al., 2017; Weber et al., 2018) are the most common forms of sporadic SVD to induce ICH. In addition, certain gene mutations are related to the severity of ICH (Devan et al., 2013; Jones et al., 2019; Tang et al., 2021).

In contrast to the significant progress that has been made in the clinical treatment of ischemic stroke, the ideal management of ICH is undetermined (Kirshner and Schrag, 2021). It is currently believed that comprehensive supportive care in the acute phase after onset is the most effective treatment of ICH patients (Hemphill et al., 2015; Parry-Jones et al., 2020). Clinical trials in the United States showed that interventional surgery or drug therapy cannot effectively reduce the mortality or morbidity of ICH (Wilkinson et al., 2018). Therefore, the risk factors that affect prognosis after cerebral hemorrhage should be comprehensively studied, especially in intensive care units (ICUs).

Doctors and scholars have been committed to investigating the risk factors that lead to the unfavorable prognosis of ICH patients. Age (Forti et al., 2016), cancer (Gon et al., 2018), infection (Lord et al., 2014), and deep vein thrombosis (Cai et al., 2021) are risk factors for the unfavorable prognosis of ICH patients. A nomogram is a graphical tool used to determine the probability of an individual experiencing a clinical event based on a statistical prediction model (Zheng et al., 2016). Nomograms that predict risk factors for mortality of ICH patients have received little attention. The purpose of our research was to develop a nomogram that can predict the 30-day mortality risk of ICH patients and thereby guide clinical practice.

## Materials and methods

### Data source

All data were extracted from the Medical Information Mart for Intensive Care III (MIMIC-III) database. MIMIC-III contains data associated with 53,423 distinct hospital admissions of adult patients (aged 16 years or older) admitted to critical care units at the Beth Israel Deaconess Medical Center in Boston, Massachusetts from 2001 to 2012 (Johnson et al., 2016). The information in the database is anonymous and therefore informed consent was not required for this study. The research personnel completed a series of courses provided by the National Institutes of Health and obtained authorization to access the MIMIC-III database after completing the required assessment (certificate number 40269495).

### Patients and variables

**FIGURE 1:**
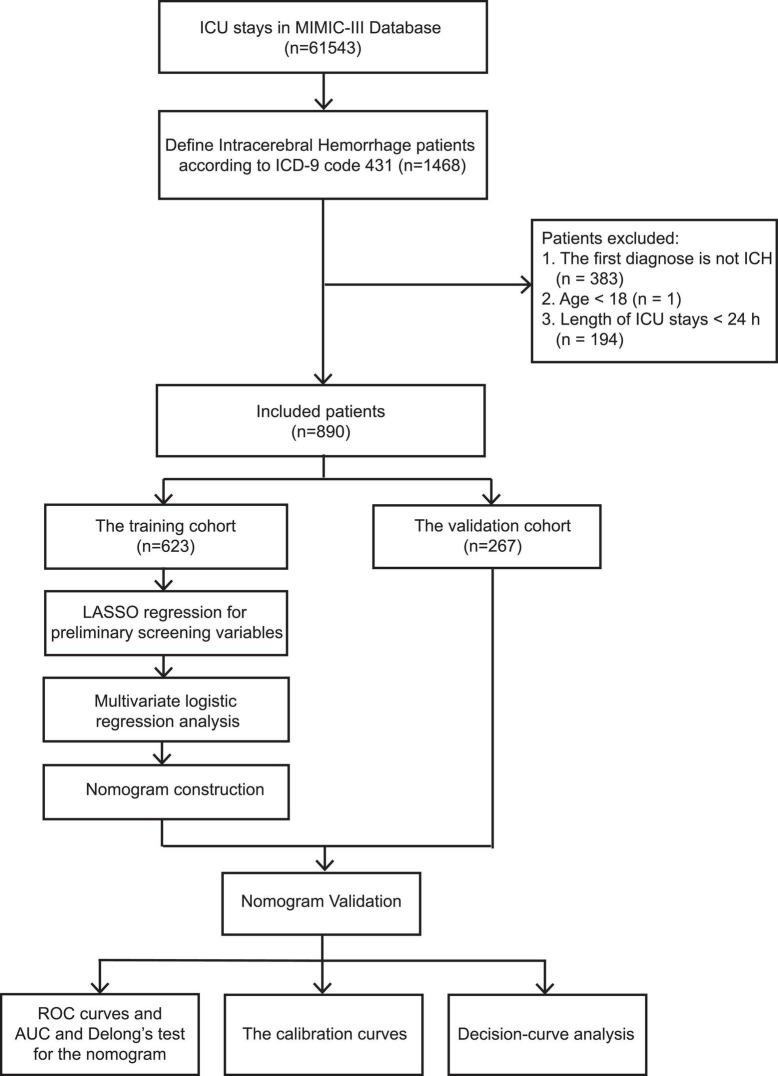
Flow diagram of the study.

The required data were extracted using the Structured Query Language in Navicat Premium (version 11.2.7.0). ICD-9 code 431 was used to extract patients diagnosed with ICH from the MIMIC-III database. The exclusion criteria were as follows: (1) first diagnosis not ICH, (2) younger than 18 years old, and (3) less than 24 h of treatment in an ICU. The flow diagram of the study is shown in Figure 1.

The icustay_id parameter was used to extract data, including age, sex, marital status, ethnicity, severity score, comorbidities, vital signs, laboratory parameters, bleeding volume, and lesion location from the corresponding table. In terms of comorbidities, chronic pulmonary diseases, hypothyroidism, renal failure, liver disease, fluid electrolyte disorders, coagulopathy, obesity, heart diseases, diabetes, other neuronal dysfunctions, and anemias were mainly extracted. The vital sign values used, including heart rate, blood pressure, respiratory rate, temperature, saturation of peripheral oxygen, glucose, and urine output, were the average values measured over 24 h before ICU admission. The laboratory results were anion gap, bicarbonate, creatinine, chloride, glucose, hematocrit, hemoglobin, platelet, and potassium levels, partial thromboplastin time, international normalized ratio, prothrombin time, blood urea nitrogen level, and white blood cell (WBC) count. Severity scoring systems included the Angus implementation of the international consensus conference definition of severe sepsis, Simplified Acute Physiology Score II (SAPSII), Sequential Organ Failure Assessment, Oxford Acute Severity of Illness Score (OASIS), Acute Physiology Score III, and Glasgow Coma Scale (GCS) score. The raw data for this study are provided as Supplementary Data 1.

The event outcome was 30-day mortality of ICH patients hospitalized in an ICU. Patients who were alive at the time of discharge were designated as survivors.

### Statistical analysis

Missing data are common in the MIMIC-III database and were explained using multiple imputations. All indicators with more than 20% missing values were eliminated. The “mice” package of R software was used to obtain ten estimated data sets. All ICH patients were randomly divided into training (70%) and validation (30%) cohorts. The former was used to build a nomogram and perform internal verification, while the latter was used to perform external verification. Categorical variables were expressed as frequency/percentage, and the chi-square test or Fisher’s exact test was used to compare different groups. The Shapiro–Wilk test was used to determine whether a continuous variable had a normal distribution. If a continuous variable was normally distributed, it was described as the mean and standard deviation. If a continuous variable was not normally distributed, it was described as the median and interquartile range.

Logistic regression was applied to determine the independent risk factors for mortality of ICH patients within 30 days. First, risk factors were identified by performing least absolute shrinkage and selection operator (LASSO) regression analysis to resolve the collinear effect (OASIS and SAPSII scores were not included as variables in subsequent studies). LASSO analysis, a method to select variables among a large number of variables for predicting outcomes, reduces the coefficient of irrelevance while retaining important variables (Zhang and Hong, 2017). The code and data set of LASSO regression are provided as Supplementary Data 1, 2. Next, different AUC values within the range of lambda were estimated using a cross-validation technique. The maximum value of lambda when the cross-validation error was within one standard error of the minimum value was chosen. In accordance with previous studies, the probability threshold for entry was 0.05 and the threshold for removal was 0.10 in this step (Xu et al., 2021). The selected variables were then subjected to multivariate logistic regression analysis in order to identify independent prognostic factors, and the results were denoted as odds ratios (ORs) and 95% confidence intervals (CIs). Finally, a nomogram was constructed based on independent prognostic factors and used to predict the 30-day mortality of ICH patients.

Subsequently, multiple indicators were used to verify the nomogram internally and externally. The area under the receiver operating characteristic curve (AUC) was utilized to evaluate the predictive ability of the contour map of the nomogram, and the AUC value was compared with those of the SAPSII and OASIS scoring systems. The statistical significance of the improvement in AUC was calculated by Delong’s test (DeLong et al., 1988). Receiver operating characteristic (ROC) curves were used to determine the optimal cut-off and its sensitivity and specificity according to the Youden index. In addition, integrated discrimination improvement (IDI) and net reclassification improvement (NRI) were applied to calculate the difference between the constructed model and the SAPSII and OASIS scoring systems for predicting the 30-day mortality of ICH patients. In addition, a calibration curve was constructed and the calibration of the nomogram was evaluated using the Hosmer–Lemeshow test. Decision curve analysis (DCA) is a widely used tool in cancer research to determine the clinical value of predictive models. Therefore, to further measure the advantages of the constructed nomogram model, DCA was performed to compare the clinical applicability of the nomogram and that of the SAPSII and OASIS scoring systems. This was achieved by calculating the net benefits for a range of threshold probabilities.

R (version 4.0.3) and SPSS (version 24.0) software were used for statistical analyses. *P* < 0.05 was considered statistically significant.

## Results

### Baseline characteristics

After applying the inclusion and exclusion criteria, 890 ICH patients were identified from the MIMIC-III database (623 and 267 in the training and validation cohorts, respectively). Length of ICU stay (2.93 [1.78, 6.34] days versus 2.89 [1.88, 7.71] days, *P* = 0.303), length of admission (8.12 [4.28, 14.44] days versus 8.60 [4.60, 15.76] days, *P* = 0.51), and mortality rate (31.9% versus 37.1%, *P* = 0.158) did not significantly differ between the two groups. In the training and validation cohorts, females accounted for 46.9 and 41.2% of ICH patients, respectively. The median ages of patients in the training and validation cohorts were 71.00 [58.00, 81.00] years and 70.00 [59.00, 80.00] years, respectively. The proportions of chronic pulmonary disease patients in the training and validation cohorts were 13.2% and 13.1%, respectively. In the training and validation cohorts, 19.9% and 23.2% of patients had complicated diabetes, respectively, while 39.0% and 36.7% of patients had another neurological insufficiency, respectively. The median SAPSII scores in the training and validation cohorts were 34.00 [27.00, 42.00] and 35.00 [28.00, 44.00], respectively. In the training and validation cohorts, the average blood glucose levels (mg/dL) were 137.00 [118.50, 164.12] and 143.00 [123.38, 166.75], respectively, while the average WBC counts (K/μL) were 10.70 [8.51, 13.57] and 10.80 [8.71, 13.50], respectively. The other baseline information is shown in Table 1.

**TABLE 1:** Patient characteristic.

Variables	Overall	Training cohort	Validation cohort	*P*
***N***	890	623	267	
**Gender** (%)				
Female	402 (45.2)	292 (46.9)	110 (41.2)	0.138
Male	488 (54.8)	331 (53.1)	157 (58.8)	
**Marital** (%)				
DSW	194 (21.8)	133 (21.3)	61 (22.8)	0.885
Married	455 (51.1)	319 (51.2)	136 (50.9)	
Single	155 (17.4)	112 (18.0)	43 (16.1)	
Unknown	86 (9.7)	59 (9.5)	27 (10.1)	
**Race** (%)				
Black	63 (7.1)	39 (6.3)	24 (9.0)	0.329
Other	198 (22.2)	138 (22.2)	60 (22.5)	
White	629 (70.7)	446 (71.6)	183 (68.5)	
**Age** (year)	71.00 [58.00, 81.00]	71.00 [58.00, 81.00]	70.00 [59.00, 80.00]	0.959
**Severe Score**				
Angus	0.00 [0.00, 1.00]	0.00 [0.00, 1.00]	0.00 [0.00, 1.00]	0.075
SAPSII	34.00 [27.25, 43.00]	34.00 [27.00, 42.00]	35.00 [28.00, 44.00]	0.265
SOFA	3.00 [2.00, 4.00]	3.00 [2.00, 4.00]	3.00 [2.00, 5.00]	0.252
OASIS	35.00 [29.00, 41.00]	35.00 [29.00, 41.00]	34.00 [29.00, 40.50]	0.877
APSIII	36.00 [26.25, 49.00]	36.00 [27.00, 50.00]	36.00 [26.00, 48.00]	0.576
GCS	14.00 [10.00, 15.00]	14.00 [10.00, 15.00]	14.00 [10.00, 15.00]	0.496
**Laboratory test**				
Anion gap (mmol/L)	14.50 [13.00, 16.19]	14.50 [13.00, 16.33]	14.00 [13.00, 16.00]	0.168
Bicarbonate (mmol/L)	24.50 [23.00, 26.63]	24.50 [23.00, 26.50]	25.00 [22.83, 26.67]	0.959
Creatinine (mg/dL)	0.90 [0.72, 1.15]	0.90 [0.70, 1.15]	0.90 [0.74, 1.16]	0.238
Chloride (mmol/L)	104.00 [101.00, 106.50]	104.00 [101.00, 106.50]	104.25 [101.59, 107.00]	0.050
Glucose (mg/dL)	138.38 [120.12, 164.73]	137.00 [118.50, 164.12]	143.00 [123.38, 166.75]	0.155
Hematocrit (g/dL)	36.53 [32.75, 39.83]	36.73 [33.11, 39.93]	35.80 [32.30, 39.50]	0.097
Hemoglobin (g/dL)	12.46 [11.20, 13.62]	12.53 [11.30, 13.60]	12.15 [10.98, 13.65]	0.129
Platelet (K/μL)	221.75 [176.62, 278.12]	224.00 [176.50, 279.75]	219.50 [177.29, 271.50]	0.374
Potassium (mmol/L)	3.89 [3.60, 4.20]	3.90 [3.61, 4.20]	3.86 [3.60, 4.15]	0.185
PTT (s)	26.40 [24.00, 29.30]	26.45 [24.10, 29.00]	26.15 [23.65, 30.03]	0.763
INR	1.13 [1.05, 1.30]	1.10 [1.05, 1.30]	1.15 [1.09, 1.35]	0.280
PT (s)	13.10 [12.50, 14.30]	13.05 [12.50, 14.20]	13.20 [12.45, 14.46]	0.429
Sodium (mmol/L)	139.67 [137.67, 141.75]	139.50 [137.50, 141.75]	140.00 [138.00, 141.71]	0.153
BUN (mmol/L)	17.00 [13.00, 22.92]	17.00 [13.33, 22.67]	17.33 [12.83, 23.00]	0.861
WBC (K/μL)	10.75 [8.55, 13.55]	10.70 [8.51, 13.57]	10.80 [8.71, 13.50]	0.781
**Vital signs**				
Heartrate (bpm)	78.86 [69.96, 89.00]	79.31 [70.02, 88.62]	77.48 [69.39, 89.54]	0.398
Systolic BP (mmHg)	134.81 [124.40, 144.22]	134.96 [124.44, 144.85]	134.46 [124.01, 143.75]	0.406
Diastolic BP (mmHg)	64.51 [57.65, 71.84]	64.36 [57.69, 71.84]	65.26 [57.59, 71.76]	0.867
Mean BP (mmHg)	85.59 [78.09, 91.91]	85.68 [77.97, 92.43]	85.08 [78.41, 91.47]	0.975
Respiratory rate (rpm)	17.72 [15.91, 19.79]	17.73 [15.86, 19.86]	17.68 [15.95, 19.71]	0.678
Temperature (°C)	36.99 [36.57, 37.43]	37.02 [36.59, 37.41]	36.96 [36.54, 37.46]	0.593
SpO_2_ (%)	98.20 [96.77, 99.25]	98.04 [96.61, 99.26]	98.37 [97.05, 99.24]	0.179
Glucose (mg/dL)	139.17 [121.06, 162.76]	138.00 [120.80, 163.40]	140.60 [123.00, 161.12]	0.665
Urine Output (mL)	1742.50 [1176.25, 2540.00]	1740.00 [1150.00, 2550.00]	1748.00 [1226.00, 2477.50]	0.880
**Comorbidities**				
**Chronic pulmonary diseases (%)**				
No	773 (86.9)	541 (86.8)	232 (86.9)	1.000
Yes	117 (13.1)	82 (13.2)	35 (13.1)	
**Hypothyroidism (%)**				
No	820 (92.1)	575 (92.3)	245 (91.8)	0.892
Yes	70 (7.9)	48 (7.7)	22 (8.2)	
**Renal failure (%)**				
No	826 (92.8)	577 (92.6)	249 (93.3)	0.843
Yes	64 (7.2)	46 (7.4)	18 (6.7)	
**Liver Diseases (%)**				
No	864 (97.1)	605 (97.1)	259 (97.0)	1.000
Yes	26 (2.9)	18 (2.9)	8 (3.0)	
**Coagulopathy (%)**				
No	828 (93.0)	584 (93.7)	244 (91.4)	0.262
Yes	62 (7.0)	39 (6.3)	23 (8.6)	
**Obesity (%)**				
No	872 (98.0)	611 (98.1)	261 (97.8)	0.959
Yes	18 (2.0)	12 (1.9)	6 (2.2)	
**Fluid-electrolyte disorders (%)**				
No	666 (74.8)	479 (76.9)	187 (70.0)	0.038
Yes	224 (25.2)	144 (23.1)	80 (30.0)	
**Heart Diseases (%)**				
No	513 (57.6)	361 (57.9)	152 (56.9)	0.836
Yes	377 (42.4)	262 (42.1)	115 (43.1)	
**Diabetes (%)**				
No	704 (79.1)	499 (80.1)	205 (76.8)	0.305
Yes	186 (20.9)	124 (19.9)	62 (23.2)	
**Other neuronal dysfunctions (%)**				
No	549 (61.7)	380 (61.0)	169 (63.3)	0.567
Yes	341 (38.3)	243 (39.0)	98 (36.7)	
**Anemias (%)**				
No	877 (98.5)	615 (98.7)	262 (98.1)	0.715
Yes	13 (1.5)	8 (1.3)	5 (1.9)	
**Bleeding Volume (cm^2^)**	10.92 [5.94, 21.74]	11.88 [6.00, 22.20]	10.08 [5.25, 19.97]	0.075
**Lesion Location (%)**				
Basal ganglia	164 (18.4)	112 (18.0)	52 (19.5)	0.104
Brain stem	5 (0.6)	3 (0.5)	2 (0.7)	
Cerebel	91 (10.2)	65 (10.4)	26 (9.7)	
Frontal	175 (19.7)	129 (20.7)	46 (17.2)	
Intraparenchymal	121 (13.6)	81 (13.0)	40 (15.0)	
Occipital	36 (4.0)	22 (3.5)	14 (5.2)	
Parietal	51 (5.7)	32 (5.1)	19 (7.1)	
Subarachnoid	23 (2.6)	15 (2.4)	8 (3.0)	
Temporal	26 (2.9)	25 (4.0)	1 (0.4)	
Thalamic	36 (4.0)	29 (4.7)	7 (2.6)	
Unknown	162 (18.2)	110 (17.7)	52 (19.5)	
**Length of ICU stays (day)**	2.93 [1.84, 6.83]	2.93 [1.78, 6.34]	2.89 [1.88, 7.71]	0.303
**Length of Admission (day)**	8.25 [4.42, 14.65]	8.12 [4.28, 14.44]	8.60 [4.60, 15.76]	0.510
**Status (%)**				
Survival	592 (66.5)	424 (68.1)	168 (62.9)	0.158
Dead	298 (33.5)	199 (31.9)	99 (37.1)	

### Nomogram construction

**FIGURE 2:**
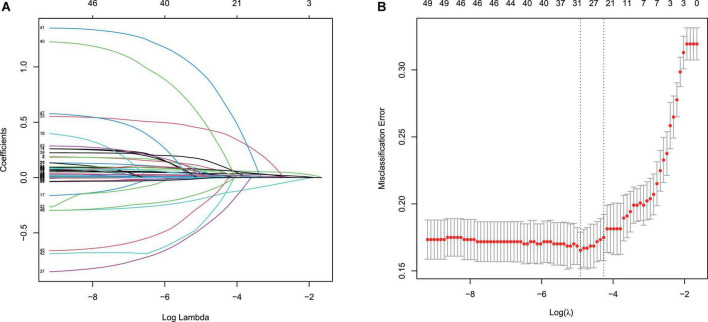
Least absolute shrinkage and selection operator (LASSO) binary logistic regression model for identifying independent risk factors for mortality in patients with ICH within 30 days. **(A)** LASSO coefficient profiles of the radiomic features. Each colored line represents the coefficient of each feature. **(B)** Plot the results of cross-validation, and the red dots in the figure represent the target parameters corresponding to each lambda. The largest lambda value is chosen when the cross-validation error is within one standard error of the minimum.

**FIGURE 3:**
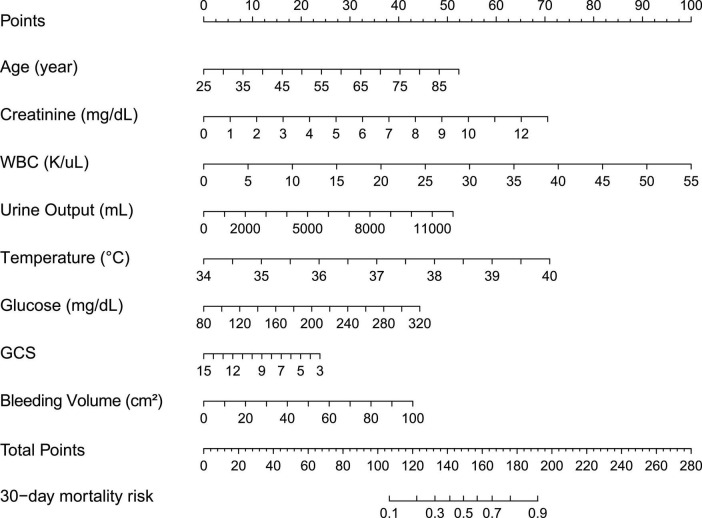
Thirty-day mortality nomogram for intracerebral hemorrhage. Nomogram included age, GCS, creatinine, WBC, temperature, glucose, urine output, and bleeding volume for predicting 30-day mortality after an acute intracerebral hemorrhage. The total point was calculated as the sum of the individual score of each of the seven variables included in the nomogram. The patient was evaluated according to each variable, and the total points were given according to the nomogram. The patient was evaluated according to the specific data of each variable, and a total score was given according to the nomogram. Based on this value, the risk of 30-day morality could be predicted.

Least absolute shrinkage and selection operator regression was applied to identify independent risk factors for mortality of ICH patients within 30 days. The different mean-squared errors within the range of log(lambda) were shown in Figure 2. When the cross-validation error was less than the standard error of the minimum value, the maximum lambda value was selected. Multivariate logistic regression identified age, GCS score, creatinine, WBC count, temperature, glucose, urine output, and bleeding volume as independent risk factors for mortality during hospitalization of ICH patients. The risk of mortality within 30 days was 1.05-fold higher for elderly patients (95% CI = 1.03–1.06). Patients with elevated WBCs at the first laboratory examination had a 1.1-fold higher risk of mortality than patients with normal results (95% CI = 1.05–1.16), while patients with a higher creatinine level had a 1.3-fold higher risk of mortality (95% CI = 1.10–1.55). Patients with hyperthermia when they entered the ICU had a 1.73-fold higher risk of mortality than patients with normal body temperature (95% CI = 1.25–2.41). Urine output (OR = 1.00, 95% CI = 1.00–1.00) and bleeding volume (OR = 1.02, 95% CI = 1.01–1.04) were also risk factors for 30-day mortality of ICH patients, while the GCS score (OR = 0.91, 95% CI = 0.86–0.95) was a protective factor (Table 2). These results were used to construct a nomogram for estimating the 30-day mortality risk of ICH patients (Figure 3).

**TABLE 2:** Factors independently associated with 30-day mortality in ICH patients.

Variables	OR	95% CI	*P*	
**Age** (year)	1.05	1.03–1.06	0.000	***
**Severe Score**				
GCS	0.91	0.86–0.95	0.000	***
**Laboratory test**				
Creatinine (mg/dL)	1.30	1.10–1.55	0.000	***
WBC (K/μL)	1.10	1.05–1.16	0.000	***
**Vital signs**				
Temperature (°C)	1.73	1.25–2.41	0.000	***
Glucose (mg/dL)	1.01	1.00–1.01	0.000	***
Urine Output (mL)	1.00	1.00–1.00	0.020	*
**Bleeding Volume** (cm^2^)	1.02	1.01–1.04	0.000	***

*P < 0.05, ***P < 0.001.

### Nomogram validation

**FIGURE 4:**
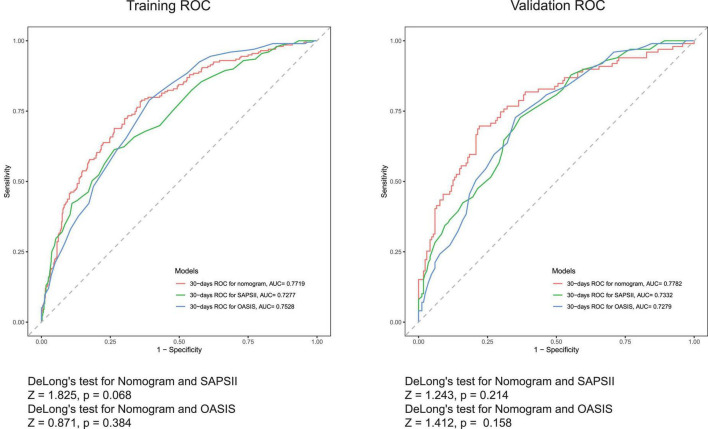
Receiver operating characteristic (ROC) curves for the SAPSII (Green), OASIS (Blue), and the nomogram (Red).

The discriminative ability of the nomogram was summarized by the ROC and Harrell’s concordance index (C-index). The C-indexes of the training and validation cohorts were 0.782 (95% CI = 0.740–0.820) and 0.778 (95% CI = 0.720–0.840), respectively. The overall predictive performance was verified using ROC curves (Figure 4). The AUCs of the nomogram were 0.772 (95% CI = 0.732–0.811) and 0.778 (95% CI = 0.719–0.838) in the training and validation cohorts, respectively. Our nomogram model had higher AUC values than SAPSII and OASIS in both the training and validation cohorts. In the training cohort, the optimal cut-off value of the nomogram was 0.276, and the sensitivity and specificity were 0.789 and 0.637, respectively. In the validation cohort, the optimal cut-off value of the nomogram was 0.403, and the sensitivity and specificity were 0.697 and 0.780, respectively. The AUCs of the individual models were further compared using Delong’s test. In the training cohort, the AUC of the nomogram did not significantly differ from those of SAPSII (*P* = 0.068) and OASIS (*P* = 0.384). In the validation cohort, our model exhibited a similar performance as SAPSII (*P* = 0.214, Delong’s test) and OASIS (*P* = 0.158, Delong’s test).

Compared with the traditional SAPSII and OASIS scoring systems, the NRI values of the nomogram were 0.257 (95% CI = –0.011–0.600) and 0.106 (95% CI = –0.082–0.405) in the training cohort, respectively, and 0.186 (95% CI = –0.192–0.670) and 0.178 (95% CI = –0.092–0.628) in the validation cohort, respectively. The corresponding IDI values were 0.048 (95% CI = 0.012–0.084), 0.028 (95% CI = –0.007–0.063), and 0.057 (95% CI = 0.001–0.112), 0.070 (95% CI = 0.018–0.123). These values indicate that our nomogram has a good discrimination ability comparable with these currently widely used scoring systems.

**FIGURE 5:**
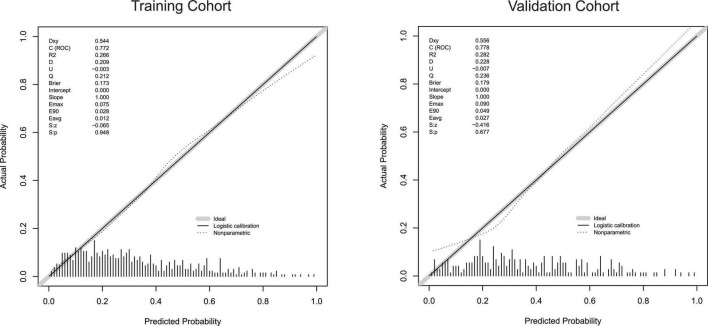
Calibration curves for the training cohort and the validation cohort.

**FIGURE 6:**
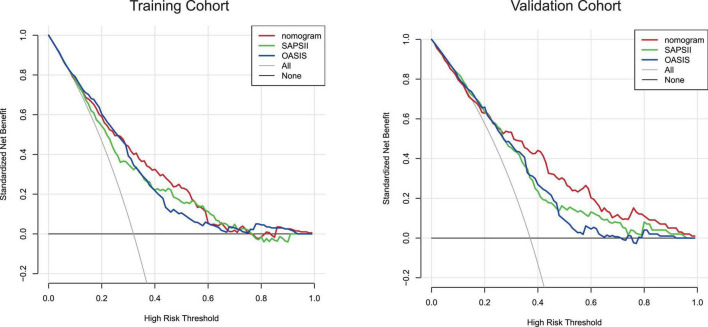
Decision-curve analysis of the training cohort and the validation cohort. Decision curve analysis depicts the clinical net benefit in pairwise comparisons across the different models. The red line indicates the nomogram, which is the model we built. The green line indicates SAPSII scoring system and the blue line indicates OASIS. Nomogram showed superior net benefit with a wider range of threshold probabilities compared with SAPSII and OASIS.

The calibration curve of the nomogram is shown in Figure 5. The calibration curves of the training and validation cohorts were both close to the diagonal. The Hosmer–Lemeshow test found no significant difference (training cohort: χ^2^ = 11.043, *P* = 0.199, validation cohort: χ^2^ = 8.643, *P* = 0.373). In summary, our nomogram provided a good simulation of the data. Finally, we drew a DCA curve to prove the clinical applicability of the nomogram and compared it with those of the SAPSII and OASIS scoring systems (Figure 6). When the threshold probability was 0.3–0.5, clinical intervention guided by our nomogram model had greater net benefit than that guided by the SAPSII and OASIS scoring systems in both cohorts.

## Discussion

In this study, we identified independent risk factors for 30-day mortality of ICH patients using LASSO and multivariate logistic regression. LASSO regression is one of the most useful tools to select features for classification and survival prognosis determination (Song et al., 2020). It can obtain fewer and more representative variable combinations from a large number of variables while avoiding model overfitting (Giral et al., 2018; Patel et al., 2018). However, LASSO regression also has shortcomings. It requires consistent initial estimates of regression coefficients, which are often unavailable in high-dimensional settings (Alhamzawi and Ali, 2018). Furthermore, the result heavily depends on the penalty parameter λ, which can lead to a very high minimization of the false discovery rate in some cases (Ternès et al., 2016).

Our results showed that age, GCS score, creatinine, WBC count, temperature, glucose, urine output, and bleeding volume were independent risk factors for 30-day mortality of ICH patients. These results were applied to construct a nomogram model for assessing the 30-day mortality risk of ICH patients. The effectiveness of the nomogram was verified by multiple indicators, including the AUC, the calibration curve, the Hosmer–Lemeshow test, IDI, NRI, and DCA. In addition, we determined the best cut-off value according to the Youden index and calculated the sensitivity and specificity. In clinical applications, the selection of a cut-off value can be based on a trade-off between misdiagnosis and missed diagnosis. Although our model had higher AUC values than the SAPSII and OASIS scoring systems, further analysis using Delong’s test did not reveal a significant difference (*P* > 0.05). However, several other indicators revealed that the predictive performance of the constructed nomogram was better than that of the SAPSII and OASIS scoring systems. These data indicate that the constructed model can reduce the number of variables and its predictive performance is not worse than that of commonly used scoring systems.

Similar to many previous studies, we found that age (Inoue et al., 2018; He et al., 2020), GCS score (Cui et al., 2020), and bleeding volume (Al-Shahi Salman et al., 2018; Du et al., 2020; He et al., 2020; Kang et al., 2021; Masotti et al., 2021; Pasi et al., 2021) were independent risk factors for the poor prognosis of ICH patients. It is generally believed that the main causes of ICH are hypertension and CAA (Schrag and Kirshner, 2020). CAA is believed to be directly related to aging (Dallaire-Théroux et al., 2022). It has also been suggested that the hematoma volume (Kuramatsu et al., 2011; Forti et al., 2016; Inoue et al., 2018) and neuroinflammation (Jiang et al., 2021; Watson et al., 2022) are more critical in elderly patients with ICH. The GCS score is the most widely used indicator for assessing the degree of coma in patients, and lower GCS scores are associated with a higher risk of death (Saika et al., 2015; Widyadharma et al., 2021). Consistent with our results, many nomogram-based studies have reported that a decrease in GCS scores significantly contributes to an increase in mortality and the likelihood of hematoma expansion in ICH patients (Yao et al., 2015; Cui et al., 2020; Chen et al., 2021; Kang et al., 2021). The bleeding volume increases upon rupture of vessels and can continue to increase after ICH is diagnosed by imaging, and, consequently, the baseline bleeding volume and hematoma enlargement are strongly associated with poor clinical outcomes (Al-Shahi Salman et al., 2018). The bleeding volume has even been reported to be the main determinant of outcomes after ICH (LoPresti et al., 2014).

Although the pathology has not been fully elucidated, ICH is accompanied by a strong inflammatory cascade, including infiltration of leukocytes, release of various inflammatory factors, and activation of microglia (Tschoe et al., 2020). Peripheral blood leukocytes are a marker of the immune response and reflect activation of the inflammatory cascade after spontaneous ICH, which causes secondary brain damage (Tapia-Pérez et al., 2016; Zhang et al., 2019). Many studies have suggested that elevated leukocyte levels are associated with worsening neurological function and increased mortality of ICH patients, consistent with our results (Lattanzi et al., 2018; Yu et al., 2019).

An elevated temperature may be caused by the inflammatory response (Iglesias-Rey et al., 2018). Up to 30–40% of ICH patients reportedly have hyperthermia and a poor prognosis (Honig et al., 2015; Liddle et al., 2022). However, in the absence of other infection signs, persistent high fever in ICH patients may be induced by perturbation of temperature control (Rabinstein and Sandhu, 2007). High fever may cause adverse events such as hematoma growth, blood-brain barrier destruction, edema, decreased cerebral blood flow, elevation of pro-inflammatory cytokines, and axon death (Balami and Buchan, 2012). Furthermore, anti-hyperthermia therapy is believed to positively affect the prognosis of ICH patients (Fischer et al., 2017; Hervella et al., 2020).

In our nomogram model, ICH patients with abnormal blood glucose levels were at increased risk of an unfavorable prognosis, consistent with other studies (Saxena et al., 2016; Wada et al., 2018; Guo et al., 2019). Elevated blood glucose levels may lead to activation of the coagulation system, inhibition of the fibrinolytic system, and production of free radicals. In addition, they may also lead to acidosis, production of excitatory amino acids, and damage of the blood-brain barrier, thereby causing secondary ischemic brain damage (Wada et al., 2018). Similar to our results, nomogram models constructed by other scholars demonstrate that an elevated blood glucose level is a risk factor for the occurrence of ICH in patients receiving thrombolytic therapy and for the poor prognosis of ICH patients (Yeo et al., 2017; Zhou et al., 2020).

Nearly one-third of hospitalized ICH patients reportedly have chronic kidney disease, possibly due to a common pathogenic mechanism and causal relationship between ICH and chronic kidney disease (Ovbiagele et al., 2014; Meier et al., 2018; Marini et al., 2020). These patients also had slightly poorer quality of care and significantly higher mortality than patients with normal renal function (Ovbiagele et al., 2014). Although the underlying mechanism remains unclear, numerous studies have found that indicators of renal insufficiency, including elevated serum creatinine levels, are independent predictors of in-hospital mortality and hematoma expansion in ICH patients (Wang and Zhang, 2017; Zhang et al., 2021).

The severity of acute kidney injury is associated with an increased serum creatinine level and decreased urine output (Kellum et al., 2015). However, we found that increased urine output was an independent risk factor for death of ICH patients. This topic has barely been previously studied. Polyuria is a very common lower urinary tract symptom in patients with neurological diseases and may involve central injury, lower urinary tract, kidney, and cardiovascular dysfunctions, and diabetes (Haddad et al., 2020). Hypothermia treatment in patients with severe traumatic brain injury may avoid the need for additional brain injury treatments that may lead to increased urine output and subsequent severe electrolyte depletion (e.g., hypophosphatemia and hypomagnesemia) (Polderman et al., 2001). In addition, increased urine output may be related to arginine vasopressin and copeptin (Christ-Crain, 2019). Plasma copeptin concentrations are reportedly significantly increased after ICH (Senn et al., 2014; Aksu et al., 2016). The increase in copeptin is correlated with the hematoma volume (Dong et al., 2011). Copeptin is directly associated with the clinical severity of ICH and poor prognostic outcomes after ICH. This may explain why ICH patients with polyuria have increased mortality (Zweifel et al., 2010; Zhang et al., 2012; Wei et al., 2014).

Although not addressed in this study, we believe that attention should be paid to parameters associated with mechanical ventilation weaning in ICH patients. Patients with severe ICH are at risk of dyspnea that requires endotracheal intubation and mechanical ventilation (Gujjar et al., 1998). However, delayed weaning may lead to increased mortality rates, complication rates, and lengths of hospital stay (Coplin et al., 2000). Traditional weaning parameters, such as the rapid shallow breathing index and negative inspiratory force, are often used as clinical indicators of mechanical ventilation (Savla et al., 2021). However, they are not always reliable in patients with brain injuries, including those who cannot achieve maximal spontaneous inspiration (dos Reis et al., 2013). Therefore, it is necessary to include indicators of optimal mechanical ventilation weaning in predictive models by logistic regression. However, in the current study, more than 20% of mechanical ventilation data were missing and this indicator was eliminated prior to analysis.

A clinical prediction model is an intuitive tool used to study the relationship between a patient’s prognosis with a specific disease and the baseline status. It helps clinicians more accurately and systematically predict the probability of a patient outcome event occurring. ICH is a stroke syndrome with a very unfavorable prognosis that widely affects the health of the public. Therefore, it is very important to build a model that can predict the mortality of ICH patients. A nomogram is a commonly used tool to evaluate oncology and medical prognosis and can graphically represent complex mathematical formulas (Balachandran et al., 2015). We reviewed several studies of nomogram prediction models with ICH patient outcomes as endpoints and found they included data on adult ICH patients (patient numbers ranging from 200 to 1000) at one or several medical centers (Cui et al., 2020; Du et al., 2020; He et al., 2020; Kang et al., 2021). All studies excluded patients with secondary ICH. Two of these studies defined the location of bleeding (Cui et al., 2020; Kang et al., 2021) and Du et al. constructed their nomogram prediction model using only imaging data (Du et al., 2020). By contrast, we did not define the location of bleeding and included it in the parameters to determine whether the bleeding at different locations affects the prognosis of patients. Furthermore, increased urine output was associated with the death of ICH patients in our model.

An advantage of our study is that the vast amount of clinical data in a public database was exploited using data mining technology. Data mining is a new field in medical research. It can build disease prediction models to assess patient risk and assist clinical decision-making by making full use of big data in public databases such as MIMIC-III and Surveillance, Epidemiology, and End Results (Yang et al., 2020; Wu et al., 2021). Numerous studies have confirmed the availability and authenticity of data in the MIMIC-III database. At the same time, possible biases in patient selection were excluded to a certain extent. In addition, the use of a large amount of data avoided errors associated with the use of fewer data, providing reliable evidence for our results.

However, our study has some limitations. Although a large number of patients were included, it was a single-center study. In addition, the included cases were mostly white therefore there may be some potential bias, and further external validation is needed to eliminate errors. In this study, we used LASSO and logistic regression in machine learning to identify variables and build a nomogram model, and did not employ other machine learning methods, such as Elastic Network Regression, Support Vector Machine, and Artificial Neural Networks. Furthermore, after screening, our model found no unexpected independent risk factors other than increased urine output. Moreover, multivariate logistic regression found that the ORs of each independent risk factor were close to 1. Further external validation using data of a subset of patients from this medical center is required to improve the confidence of the results. Nevertheless, we believe that the proposed model may contribute to further understanding of the mortality of ICH patients.

## Conclusion

The study identified independent risk factors for 30-day mortality in ICH patients and used them to construct a predictive nomogram model. The results of our study may provide support for improving the prognosis of ICH patients.

## Relevance for clinical practice

We developed a nomogram model to predict the 30-day mortality in patients with ICH based on baseline characteristics, laboratory tests, and imaging data of ICH patients admitted to the ICU from the MIMIC-III database. Compared with the SAPSII and OASIS systems, the nomogram has comparable performance in predicting mortality of patients with ICH. It’s more concise and easier to use, thus providing a new reference guideline for the treatment and care of patients with ICH.

## Data availability statement

The original contributions presented in this study are included in the Supplementary data sheet 1, further inquiries can be directed to the corresponding authors. The original data are available on the MIMIC-III website at https://mimic.physionet.org/, http://dx.doi.org/10.13026/C2XW26.

## Ethics statement

The study was an analysis of a third-party anonymized publicly available database with pre-existing institutional review board (IRB) approval. Data extracted from the MIMIC III database do not require individual informed consent because MIMIC III database research data is publicly available, and all patient data are de-identified.

## Author contributions

MT conceived the study. JZ created the study protocol, performed the statistical analyses, and wrote the first manuscript draft. HC conceived the study and critically revised the manuscript. CL assisted with the study design and performed the data collection. ZC, JY, YZ, and SL assisted with study coordination and helped draft the manuscript. HL assisted with manuscript revision and data confirmation. MT contributed to data interpretation and manuscript revision. All authors read and approved the final manuscript.

## Supplementary material

Supplementary Data 1 Raw data.

Supplementary Data 2 Code for LASSO analysis.
