# Supplementary material for: Development and validation of a nomogram to predict the 30-day mortality risk of patients with intracerebral hemorrhage
Source: Front Neurosci. 2022 Aug 10;16:942100. doi: 10.3389/fnins.2022.942100 (PMC9400715; doi:10.3389/fnins.2022.942100)
Supplement: Supplementary Data 2 — Code for LASSO analysis. [file Data_Sheet_2.pdf]

```
library('glmnet')

# split the data into development cohort
seerd <- sample(nrow(row_data),0.7*nrow(row_data))
cohort.dev <- row_data[seerd,];dim(cohort.dev)
dataInput <- cohort.dev

# define the features and target
x<- data.matrix(dataInput[,c(2:51)])
y<- data.matrix(dataInput$status)

# plot the Figure 2A for Coefficients
f1 = glmnet(x, y, family="binomial", nlambda=100, alpha=1)
plot(f1, xvar="lambda", label=TRUE)
dev.off()

# plot the Figure 2B for Misclassification Error and variable selection
alpha1.fit <- cv.glmnet(x,y,type.measure = "class",alpha=1,family="binomial") ## type.measure = "class"
plot(alpha1.fit)
dev.off()

# get the variables that screened
#print(alpha1.fit)
lasso_coef <- as.data.frame(as.matrix(coef(f1,s=alpha1.fit$lambda.1se)))
factorsInput <- row.names(lasso_coef)[lasso_coef$`1` != 0][-1];
```
